# Aerosol immunization with influenza matrix, nucleoprotein, or both prevents lung disease in pig

**DOI:** 10.1038/s41541-024-00989-8

**Published:** 2024-10-13

**Authors:** Eleni Vatzia, Basudev Paudyal, Barbara Dema, Brigid Veronica Carr, Ehsan Sedaghat-Rostami, Simon Gubbins, Bhawna Sharma, Elliot Moorhouse, Susan Morris, Marta Ulaszewska, Ronan MacLoughlin, Francisco J. Salguero, Sarah C. Gilbert, Elma Tchilian

**Affiliations:** 1 The Pirbright Institute, Pirbright, UK; 2 Nuffield Department of Medicine, University of Oxford, Oxford, UK; 3 Aerogen Ltd., Galway, Ireland; 4 United Kingdom Health Security Agency, UKHSA-Porton Down, Salisbury, UK

**Keywords:** Influenza virus, Vaccines

## Abstract

Current influenza vaccines are strain-specific and require frequent updates to combat new strains, making a broadly protective influenza vaccine (BPIV) highly desirable. A promising strategy is to induce T-cell responses against internal proteins conserved across influenza strains. In this study, pH1N1 pre-exposed pigs were immunized by aerosol using viral vectored vaccines (ChAdOx2 and MVA) expressing matrix (M1) and nucleoprotein (NP). Following H3N2 challenge, all immunizations (M1, NP or NPM1) reduced lung pathology, but M1 alone offered the greatest protection. NP or NPM1 immunization induced both T-cell and antibody responses. M1 immunization generated no detectable antibodies but elicited M1-specific T-cell responses, suggesting T cell-mediated protection. Additionally, a single aerosol immunization with the ChAdOx vaccine encoding M1, NP and neuraminidase reduced lung pathology. These findings provide insights into BPIV development using a relevant large natural host, the pig.

## Introduction

Current vaccine strategies against influenza viruses induce strain-specific neutralizing antibodies but the rapid emergence of variant strains leads to loss of protection, necessitating frequent updating of vaccines. An alternative approach is to target conserved antigens that induce CD4+ and CD8+ T-cell responses, providing the basis for a broadly protective influenza vaccine (BPIV). Furthermore, although seasonal influenza vaccines are administered parenterally, administration of vaccines to the respiratory tract is a powerful route to control respiratory pathogens and has been shown to be highly effective in inducing broad protection^1–5^. The presence of protective immune responses in the lung is particularly important as severe influenza is due to lung infection, and highly pathogenic avian influenza viruses have tropism for the lower respiratory tract.

The protective role of CD4+ and CD8+ T-cell responses in humans has been demonstrated in experimental influenza challenge studies^6,7^. Community cohort studies have further confirmed the association between T-cell responses against conserved internal proteins particularly polymerase binding protein 1 (PB1), nucleoprotein (NP), matrix 1 (M1) and reduced viral shedding and severity of disease^8,9^. Since most individuals have been repeatedly exposed to influenza viruses, they already have T-cell responses to the conserved influenza core proteins and therefore, immunization strategies aiming to boost these T-cell immune responses might be highly beneficial^10^. Immunization by both the replication deficient chimpanzee adenovirus ChAdOx and the replication deficient modified vaccinia virus Ankara vaccine (MVA) can boost M1- and NP-specific T-cell responses as demonstrated in clinical trials with young and older adults^11^. The inclusion of neuraminidase (NA) in the vaccines can further provide more robust and broad protection as although NA sequences do evolve over time, this occurs more slowly than for hemagglutinin (HA)^12,13^ and immunization with recombinant NA protein has been shown to induce protective immunity in different animal models, especially when the antigen is given mucosally^14–19^.

We have previously demonstrated that the viral vectored vaccines ChAdOx2 and MVA expressing NP, M1 and NA2 are highly protective in pigs^20^. Pigs are a relevant natural large animal model to study immunity to influenza^3,21–23^. They have many genetic, physiological, anatomical and immunological similarities to humans and are infected by similar subtypes of influenza viruses in particular H1N1pdm09 (pH1N1)^23–27^. We used pH1N1 pre-exposed pigs to mimic the situation in humans who are repeatedly exposed to numerous influenza viruses and immunized them sequentially by aerosol with ChAdOx2-NPM1-NA2 and MVA-NPM1-NA2 in order to reach the whole respiratory tract^20^. Immunization abolished viral shedding and lung pathology following H3N2 virus challenge. However, as the NA2 was matched to the challenge H3N2 virus we were not able to determine the contribution of the T-cell and antibody responses in protection, nor could the contribution of the individual antigens be assessed. Here we explored whether a single immunization with ChAdOx-NPM1-NA2 would provide protection in pH1N1 pre-exposed pigs, a more easily deployable strategy in clinical practice. Additionally, we investigated whether both NP and M1 antigens are required for the observed protective effect.

## Results

### Immunogenicity and efficacy of single aerosol immunization with ChAdOx2-NPM1-NA2 in pH1N1 pre-exposed pigs

**Fig. 1:**
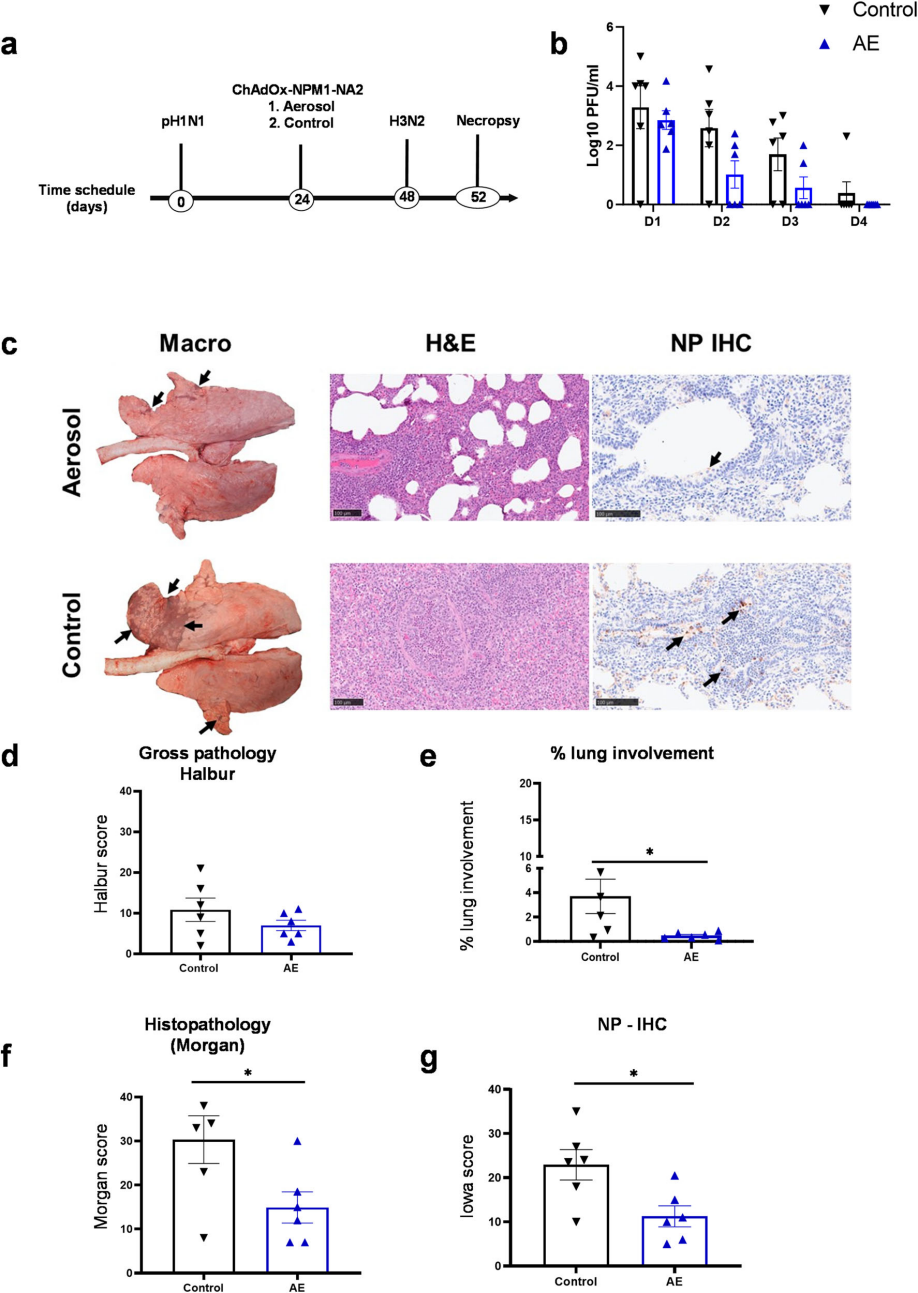
Experimental design, viral shedding and lung pathology following single-dose aerosol immunization with ChAdOx-NPM1-NA2. **a** Twelve pigs were infected with pH1N1 and 24 days later one group of six pigs were immunized with ChAdOx-NPM1-NA2 by aerosol (AE). After 28 days they were infected with H3N2 and after 4 days were culled. Control (C) animals were infected but not immunized. **b** Virus load within the upper respiratory tract was determined by plaque assay in daily nasal swabs post H3N2 infection (D1–D4). **c** Representative lung gross pathology, histopathology (H&E staining) and immunohistochemical (IHC) NP staining of each group. Gross pathology is observed as areas of consolidation (arrows). Bronchiolo- and broncho-interstitial pneumonia with necrosis of epithelial cells and inflammatory infiltrates in the airways and parenchyma are observed with higher severity in the control group. Virus NP is detected by IHC (brown stain, arrows) within the bronchiolar wall and luminae and occasionally within the parenchyma. **d** Gross lesion scores, **e** percentage of lung surface with lesions using image analysis, **f** histopathology and **g** IHC NP scores (“Iowa”) are shown. The top of each bar indicates the mean and the line the SEM. Each symbol represents one animal. Asterisks denote significance between indicated groups (*p < 0.05) and were analyzed either by one-way ANOVA and Bonferroni’s multiple comparisons test when the data were normally distributed (b) or with Kruskal–Wallis and Dunn’s multiple comparisons test when normality was not achieved (**d**–**g**). Bar = 100 µm.

Following the demonstration of the protective efficacy of heterologous prime-boost immunization with ChAdOx-NPM1-NA2 and MVA-NPM1-NA2^20^, we next investigated whether a single ChAdOx-NPM1-NA2 aerosol immunization would provide protection. Twelve pigs were inoculated intranasally with pH1N1, and viral shedding assayed by plaque assay, confirming the infection of all 12 animals (Supplementary Fig. 1). Twenty-four days after the pH1N1 exposure, pigs were randomly divided into two groups of six pigs. One group were immunized by aerosol (AE) with ChAdOx2-NPM1-NA2 using a vibrating mesh nebulizer and the second group were unimmunized controls. Four weeks later all pigs were infected intranasally with H3N2, daily nasal swabs were collected to investigate viral shedding and 4 days later the animals were humanely euthanized (Fig. 1a).

The immunized animals showed a reduction in H3N2 viral shedding in daily nasal swabs at days 2 and 3 post challenge, although this did not reach statistical significance (*p* = 0.058 and *p* = 0.099, respectively) (Fig. 1b). No virus was detected in the BAL and lung of either group (data not shown). Representative lung gross pathology, histopathology and immunohistochemical NP staining are shown in Fig. 1c. Gross pathology was observed as areas of consolidation mostly affecting the cranial and medial lobes^28^. No significant difference in gross lung pathology was found between the immunized and control groups, although the percentage of lung lesion calculated with image analysis of the dorsal and ventral views of the lungs showed a significantly higher score in the control compared to the aerosol group (Fig. 1d, e). The histopathological analysis revealed significantly higher scores in the control than the aerosol immunized animals (Fig. 1f). Labeling of influenza NP by immunohistochemistry (NP-IHC) was observed in the bronchiolar and bronchial epithelia, cell debris and inflammatory cells within the airways luminae and occasional inflammatory cells within the lung parenchyma (Fig. 1c). The NP-IHC staining was significantly higher in the control group in comparison to the immunized group, as evaluated within the “Iowa” scoring system^29^ (Fig. 1g)

We also assessed antibody and T-cell responses. Circulating (serum) virus-specific IgG against pH1N1, H3N2 and recombinant NA2 from H3N2 (Supplementary Fig. 2a–c) and local (BAL) virus-specific IgG and IgA against pH1N1 and H3N2 (Supplementary Fig. 2d–g) were measured by ELISA. The immunized animals showed higher IgG responses in serum against pH1N1, H3N2 and recNA2 post vaccination compared to the control group, although statistical significance was not reached (Supplementary Fig. 2a–c). Similarly, no significant differences were observed in BAL antibody responses between immunized and control animals, although a trend for higher titers of IgG H1N1, H3N2 and IgA H3N2 was detected in the aerosol group. The IFNγ response following H3N2 challenge was evaluated by ELISpot in BAL (Supplementary Fig. 2h–l). A trend for higher number of IFNγ producing cells following stimulation with M1 (*p* = 0.06) and pH1N1 (*p* = 0.07) was observed in the immunized animals compared to controls, but this did not reach statistical significance (Supplementary Fig. 2i, k).

Taken together these data suggest that a single aerosol administration of ChAdOx2-NPM1-NA2 significantly reduced the area of lung lesions and lung inflammation assessed by histopathology with a trend toward reduced viral shedding at days 2 and 3. This was correlated with increased antibody and T-cell responses in the immunized animals, although the changes were not statistically significant. Since we know that only 30% of the aerosol dose is deposited in the pig^30^, it may be that a single immunization with a higher dose would improve protection to the level achieved by prime boosting.

### Generation of single antigen vaccines and immunogenicity in mice

To determine the contribution of NP and M1 antigens for the induction of protective immune responses we generated ChAdOx2 and MVA vectored vaccines expressing NP only, M1 only or an NPM1 fusion protein and tested their immunogenicity in mice. Mice were immunized either intramuscularly (IM) or intranasally (IN) to stimulate intrapulmonary response as observed with aerosol immunization in pigs. Mice were vaccinated with 10^8^ IU of ChAdOx-NP or ChAdOx-M1 or ChAdOx-NPM1 and after 4 weeks boosted with 10^6^ PFU MVA-NP, MVA-M1 or MVA-NPM1 using the same antigen and route. Mice were culled 4 weeks later. IFNγ ELISpot responses were measure in fresh splenocytes following stimulation with NP (Supplementary Fig. 3a) and M1 (Supplementary Fig. 3b) pools of overlapping peptides covering the NP and M1 proteins included in the vaccines. The frequencies of CD4+ and CD8 + IFNγ/TNF double secreting cells in fresh splenocytes and lung cells were also measured by intracellular cytokine staining after NP (Supplementary Fig. 3c, d, g) and M1 (Supplementary Fig. 3e, f, h) stimulation. The gating strategy is shown in Supplementary Fig. 4a.

Intramuscular immunization induced higher splenic immune responses compared to intranasal immunization, although this was significant only for the M1 (Supplementary Fig. 3b, e, f) and NPM1 groups (Supplementary Fig. 3a, c, d). Significant reduction in M1-specific responses were detected in NPM1 immunized animals compared to M1 group (Supplementary Fig. 3b, f). No differences in responses were detected between NP and NPM1 immunization following NP stimulation (Supplementary Fig. 3a, c, d).

Lung immune responses following intranasal immunizations resulted in CD8+ IFNγ TNF producing cells specific to NP and M1 antigens (Supplementary Fig. 3h), which were also tissue resident memory cells as defined by their CD103 and CD69 expression (Supplementary Figs. 3i and 4b). M1-specific responses were significantly reduced in the NPM1 group compared to M1 (Supplementary Fig. 3h) while no differences in NP-specific responses between NP and NPM1 immunized animals were observed.

These data indicate that the ChAdOx2 and MVA vaccines were immunogenic in mice, inducing both systemic and local lung CD4+ and CD8+ cytokine producing cells.

### Protection following prime-boost immunization with single NP, single M1 and NPM1 in pH1N1 pre-exposed pigs

**Fig. 2:**
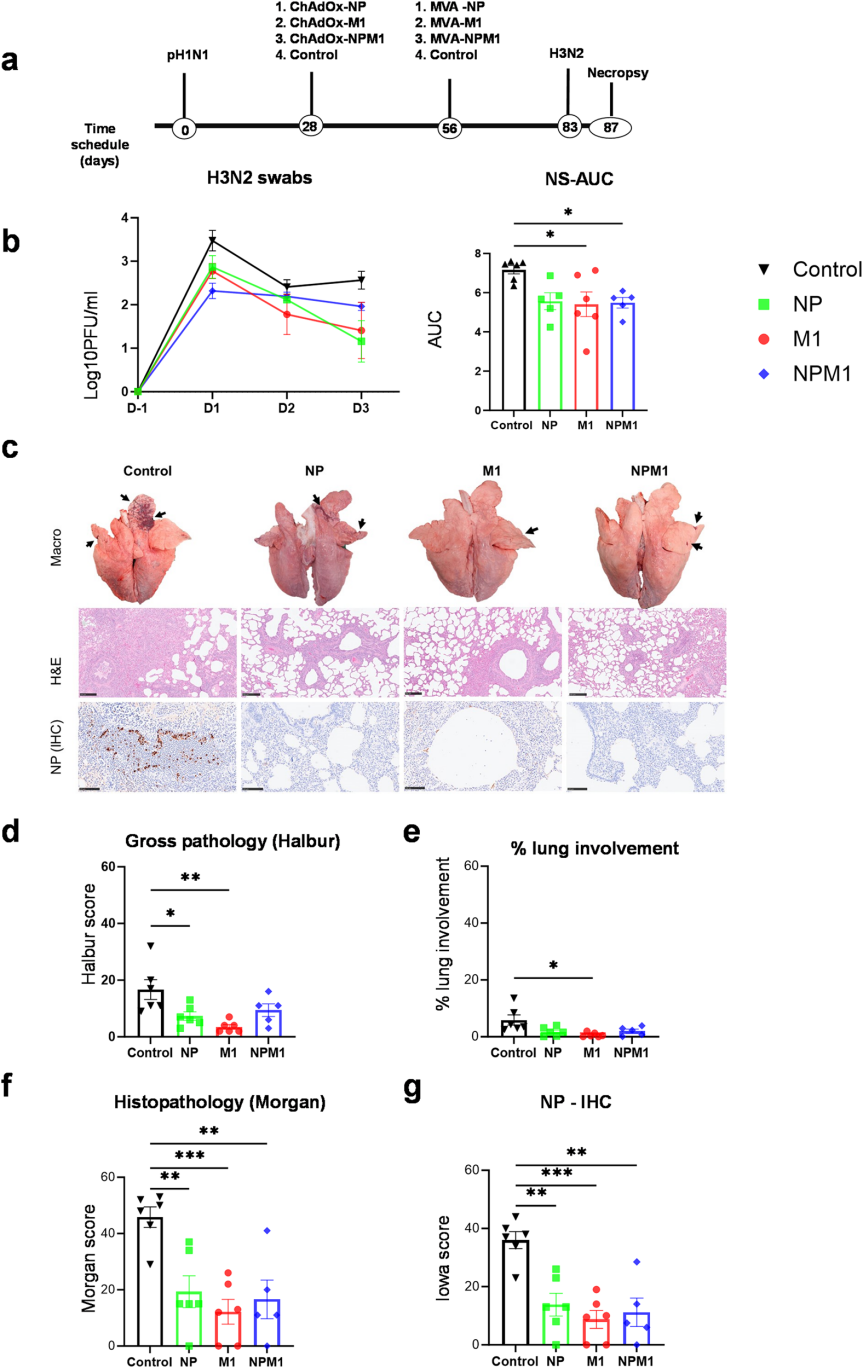
Experimental design, viral shedding and lung pathology following prime-boost immunizations with NP, M1 and NPM1 in pH1N1 pre-exposed pigs. **a** Twenty-four pigs were infected with pH1N1 and 4 weeks later groups of six pigs were immunized by aerosol with ChAdOx-NP, ChAdOx-M1 or ChAdOx-NPM1. After further 4 weeks they were boosted by aerosol with MVA including the same antigens. Four weeks after the MVA boost all pigs were infected with H3N2 virus and after 4 days pigs were culled. Control (C) animals were infected but not immunized. **b** Virus load within the upper respiratory tract was determined by plaque assay in daily nasal swabs post H3N2 infection (D1–D4). Significant differences in nasal shedding determined by the area under the curve (AUC). **c** Representative lung gross pathology, histopathology (H&E staining) and immunohistochemical (IHC) NP staining of each group. Gross pathology is observed as areas of consolidation (arrows). Bronchiolo- and broncho-interstitial pneumonia with necrosis of epithelial cells and inflammatory infiltrates in the airways and parenchyma are observed with higher severity in the control group. Virus NP is detected by IHC (brown stain) within the bronchiolar wall and luminae and occasionally within the parenchyma. **d** Gross lesion scores, **e** percentage of lung surface with lesions using image analysis, **f** histopathology and **g** IHC NP scores (“Iowa”) are shown. The top of each bar indicates the mean and the line the SEM. Each symbol (circle, square, diamond and triangle) represents one animal. Asterisks denote significance between indicated groups (*p < 0.05, **p < 0.01, ***p < 0.001) and were analyzed either by one-way ANOVA and Bonferroni’s multiple comparisons test when the data were normally distributed (**b**, **d**–**g**). Bar = 100 µm.

We next wished to determine the contribution of NP and M1 antigens in the induction of protective immune responses, in the pre-exposed pig model. Twenty-four pigs were infected with pH1N1 and as with the previous study, nasal shedding was detected in all animals confirming successful infection (Fig. 2a and Supplementary Fig. 5). Four weeks later, the animals were randomly divided into four groups of six pigs and immunized by aerosol using a vibrating mesh nebulizer with either ChAdOx2-NPM1, ChAdOx2-NP or ChAdOx2-M1. Six pH1N1-exposed but unimmunized pigs were used as controls. Four weeks later, the three immunized groups were boosted by aerosol with an MVA vaccine including the same antigens NPM1, NP or M1 as in the first ChAdOx2 immunization. Four weeks after the MVA boost all pigs were infected intranasally with H3N2. Animals were humanely culled 4 days later, and tissues collected for virological, pathological, and immunological analyses (Fig. 2a).

Viral shedding was analyzed in nasal swabs by plaque assay (Fig. 2b). There was a significant decrease in viral shedding post H3N2 infection in the M1 and NPM1 immunized animals as assessed by the area under the curve. As in the previous experiment, no virus was detected in BAL and lungs, most likely because the virus was too diluted following the washing of the lungs.

The control group showed significant gross and histopathological lesions as well as NP-IHC staining within the airway epithelium and inflammatory cell infiltrates in the airways and parenchyma (Fig. 2c). The immunized groups showed lower gross pathology score, with statistical significance for the NP and M1 groups (Fig. 2d, e). However, there was a significant reduction of histopathological scores for all the immunized groups compared to the controls, including the “Iowa” score which considers NP staining, with the M1 group showing the lowest scores^29,31^ (Fig. 2f, g). These results indicate that immunization with either NP or M1 is sufficient to reduce lung pathology and viral shedding following heterologous H3N2 virus challenge in pH1N1 pre-exposed pigs and that there is no benefit in including both NPM1 antigens. In all measures of lung involvement M1 alone showed the greatest reduction compared to controls but there was no statistically significant difference between M1, NP and NPM1 immunized animals.

### Antibody responses following prime-boost immunization with single NP, single M1 and NPM1 in pH1N1 pre-exposed pigs

**Fig. 3:**
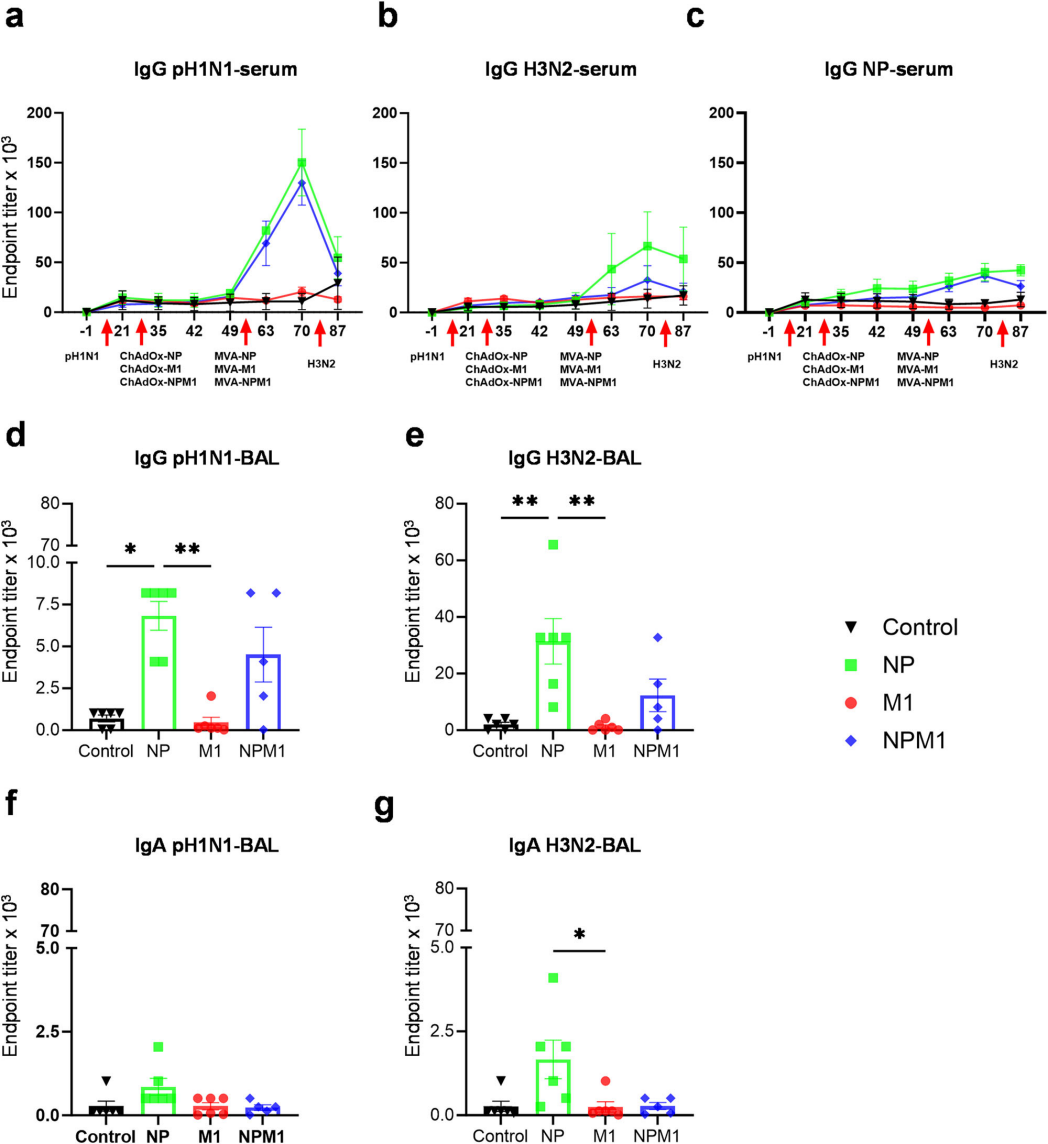
Antibody responses following prime-boost immunization with NP, M1 and NPM1 in pH1N1 pre-exposed pigs. **a** pH1N1, **b** H3N2 and **c** NP-specific IgG responses in serum were determined by ELISA at the indicated time points. **d** pH1N1, **e** H3N2-specific IgG and **f** pH1N1 and **g** H3N2 IgA responses in BAL, were determined by ELISA 4 days after H3N2 challenge. The mean and standard error (SEM) is presented in each time point (**a**–**g**). The arrows below D0, D28, D56 and D83 indicate the time of pH1N1 challenge, ChadOx immunizations, MVA boost and H3N2 challenge respectively. Significant differences in antibody responses in serum are listed in Table 1. The top of each bar indicates the mean, and the line indicates the standard error mean (SEM). Each symbol (circle, square, diamond and triangle) represents one animal. Asterisks denote significance between indicated groups (*p < 0.05, **p < 0.01) and were analyzed either by one-way ANOVA and Bonferroni’s multiple comparisons test when the data were normally distributed (**a**–**c**, **e**) or with Kruskal–Wallis and Dunn’s multiple comparisons test when normality was not achieved (**d**, **f**, **g**).

Serum IgG against pH1N1, H3N2 and recombinant NP (Fig. 3a–c) and BAL virus-specific IgG and IgA against pH1N1 and H3N2 (Fig. 3d–g) were measured by ELISA. The NP immunized group had significantly higher serum pH1N1, H3N2 and NP IgG titers after the MVA boost in comparison to the control and M1 groups (Table 1 and Fig. 3a–c). Similarly, NPM1 immunization induced higher pH1N1, H3N2 and NP IgG titers compared to control and M1 groups (Table 1). M1 immunization did not boost antibody responses, which were comparable to those in the pH1N1 pre-exposed unimmunized control group. In BAL (Fig. 3d–g), NP immunization induced significantly greater IgG-pH1N1, IgG-H3N2 and IgA-H3N2 titers than M1 and control groups. Antibody titers in NPM1 immunized group, although higher than the control and M1 groups, were not statistically significantly different. No differences in pH1N1-specific IgA responses were detected between the groups.

**Table 1:** Significant differences in ELISA and ELISpot responses between NP, M1, NP/M1 and control groups at different time points after immunization

Assay	Stimulus	Significances identified between groups post immunization					
		Day 35	Day 42	Day 49	Day 63	Day 70	Day 87 (PM)
ELISA IgG (Serum)	H1N1		No significance (*P* > 0.05)	No significance (*P* > 0.05)	NP > M1 (*P* < 0.0001) NP > Control (*P* < 0.0001) NPM1 > M1 (*P* < 0.0001) NPM1 > Control (*P* < 0.0001)	NP > M1 (*P* < 0.0001) NP > Control (*P* < 0.0001) NPM1 > M1 (*P* < 0.0001) NPM1 > Control (*P* < 0.0001)	
	H3N2		No significance (*P* > 0.05)	No significance (*P* > 0.05)	NP > M1 (*P* = 0.002) NP > NPM1 (*P* = 0.008) NP > Control (*P* = 0.0004)	NP > M1 (*P* < 0.0001) NP > NPM1 (*P* = 0.0002) NP > Control (*P* < 0.0001)	NP > M1 (*P* < 0.0001) NP > NPM1 *(P* = 0.001) NP > Control (*P* < 0.0001)
	NP		NP > M1 (*P* = 0.0002) NP > Control (*P* = 0.01)	NP > M1 (*P* = 0.0002) NP > Control (*P* = 0.01)	NP > M1 (*P* < 0.0001) NP > Control (*P* < 0.0001) NPM1 > M1 (*P* < 0.0001) NPM1 > Control (*P* = 0.0002)	NP > M1 (*P* < 0.0001) NP > Control (*P* < 0.0001) NPM1 > M1 (*P* < 0.0001) NPM1 > Control (*P* < 0.0001)	NP > M1 (*P* < 0.0001) NP > NP/M1 (*P* = 0.0002) NP > Control (*P* < 0.0001) NPM1 > M1 (*P* = 0.0007)
ELISpot (PBMC)	H1N1	No significance (*P* > 0.05)	No significance (*P* > 0.05)	No significance (*P* > 0.05)	No significance (*P* > 0.05)	No significance (*P* > 0.05)	NP > M1 (P = 0.0470) NP > NPM1 (*P* = 0.0010) NP > Control (*P* = 0.006)
	H3N2	No significance (*P* > 0.05)	No significance (*P* > 0.05)	No significance (*P* > 0.05)	No significance (*P* > 0.05)	No significance (*P* > 0.05)	NP > M1 (*P* < 0.0001) NP > NPM1 (*P* < 0.0001) NP > Controls (*P* = 0.0003)
	M1	M1 > NP (*P* = 0.04) M1 > Controls (*P* = 0.034)	No significance (*P* > 0.05)	No significance (*P* > 0.05)	M1 > NP (*P* = 0.0006) M1 > NPM1 (*P* = 0.018) M1 > Controls (*P* = 0.008)	No significance (*P* > 0.05)	No significance (*P* > 0.05)
	NP	No significance (*P* > 0.05)	No significance (*P* > 0.05)	No significance (*P* > 0.05)	NP > Controls (*P* = 0.046)	No significance (*P* > 0.05)	NP > M1 (*P* < 0.0001) NP > NPM1 (*P* = 0.0001) NP > Controls (*P* < 0.0001)

In summary, NP immunization boosted both serum and BAL antibody responses, while NPM1 boosted only the serum responses. M1 immunization did not boost virus- or M1-specific antibody response, suggesting that the antibody responses in the NPM1 immunized animals are due to the NP component.

### T-cell responses following prime-boost immunization with single NP, single M1 and NPM1 in pH1N1 pre-exposed pigs

**Fig. 4:**
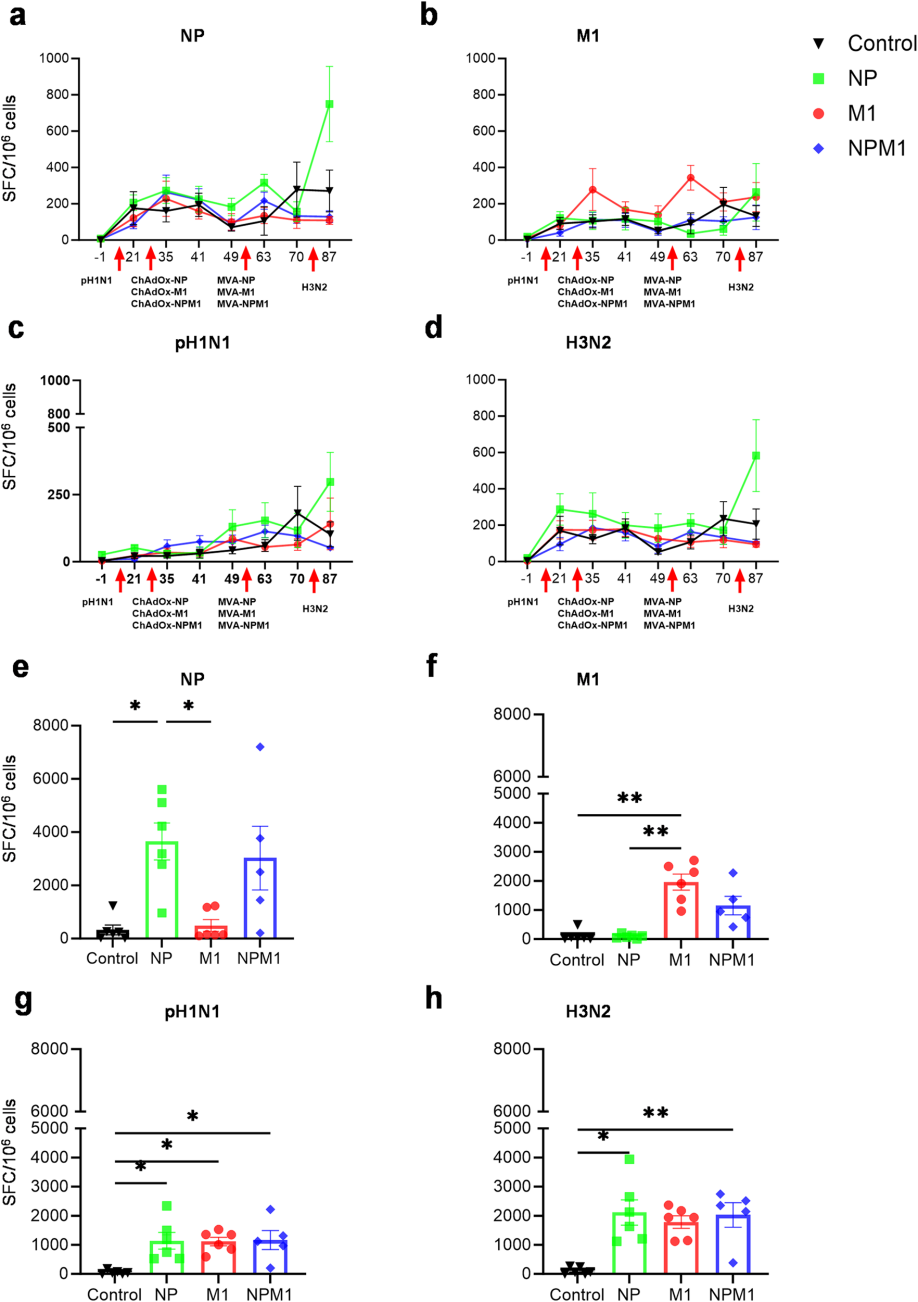
IFNγ ELISpot responses in PBMC and BAL after immunization with NP, M1 and NPM1. IFNγ secreting spot forming cells (SFC) in blood (**a**–**e**) and BAL (**e**–**h**) were enumerated following stimulation with a pool of peptides covering NP (**a**, **f**) and M1 (**b**, **g**) proteins or pH1N1 (**c**, **g**) and H3N2 (**d**, **h**) viruses. The red arrows indicate the time of pH1N1 challenge, immunizations with ChAdOx and MVA, and H3N2 challenge. Significant differences are listed in Table 1. Each symbol (circle, square, diamond and triangle) represents an individual animal, the top of the bar the mean and the line the standard error (SEM). Asterisks denote significance between indicated groups (*p < 0.05, **p < 0.01) and were analyzed either by one-way ANOVA (**a–d**) and Bonferroni’s multiple comparisons test (**g**) when the data were normally distributed or with Kruskal–Wallis and Dunn’s multiple comparisons test when normality was not achieved (**e**, **f**).

IFNγ ELISpot analysis was performed to quantify IFNγ producing cells in PBMC (Fig. 4a–d) and BAL (Fig. 4f–h) following stimulation with either pH1N1 and H3N2 live viruses or with pools of overlapping peptides covering the NP and M1 proteins included in the vaccines. M1 immunization significantly increased the M1-specific IFNγ responses 1 week after the prime with ChAdOx2-M1 and 1 week after the MVA-M1 boost (Fig. 4b and Table 1). NP-specific responses were significantly greater in the NP immunized animals 1 week after the boost (Fig. 4a), but not in the NPM1 group. On the last day of the study (D87), the NP immunized group exhibited the highest pH1N1, H3N2- and NP-specific IFNγ response of all the groups (Fig. 4a, c, d and Table 1).

As in blood, the NP-specific IFNγ responses in the BAL were significantly higher in the NP immunized group compared to the control and M1 groups (Fig. 4e). M1 immunization induced the highest M1-specific IFNγ response compared to control and NP groups (Fig. 4f). Following pH1N1 stimulation all immunized groups exhibited a significantly higher number of IFNγ producing BAL cells than the control (Fig. 4g), while H3N2-specific cells were significantly greater in the NPM1, and NP immunized groups compared to control (Fig. 4h). ChAdOx2 immunization did not significantly boost blood antibody or T-cell responses after pH1N1 pre-exposure (Figs. 3a–c and 4a–d). However, our previous studies have shown a strong boosting effect of ChAdOx2-NP/M1-NA2 and ChAdOx2-NP/M1-NA2 /MVA-NP/M1-NA2 in the BAL^20,32^ so that it is likely that respiratory tract immune responses are increased by the aerosol immunization used in the present study. We did not assess this, because it would have necessitated culling additional animals prior to influenza challenge.

**Fig. 5:**
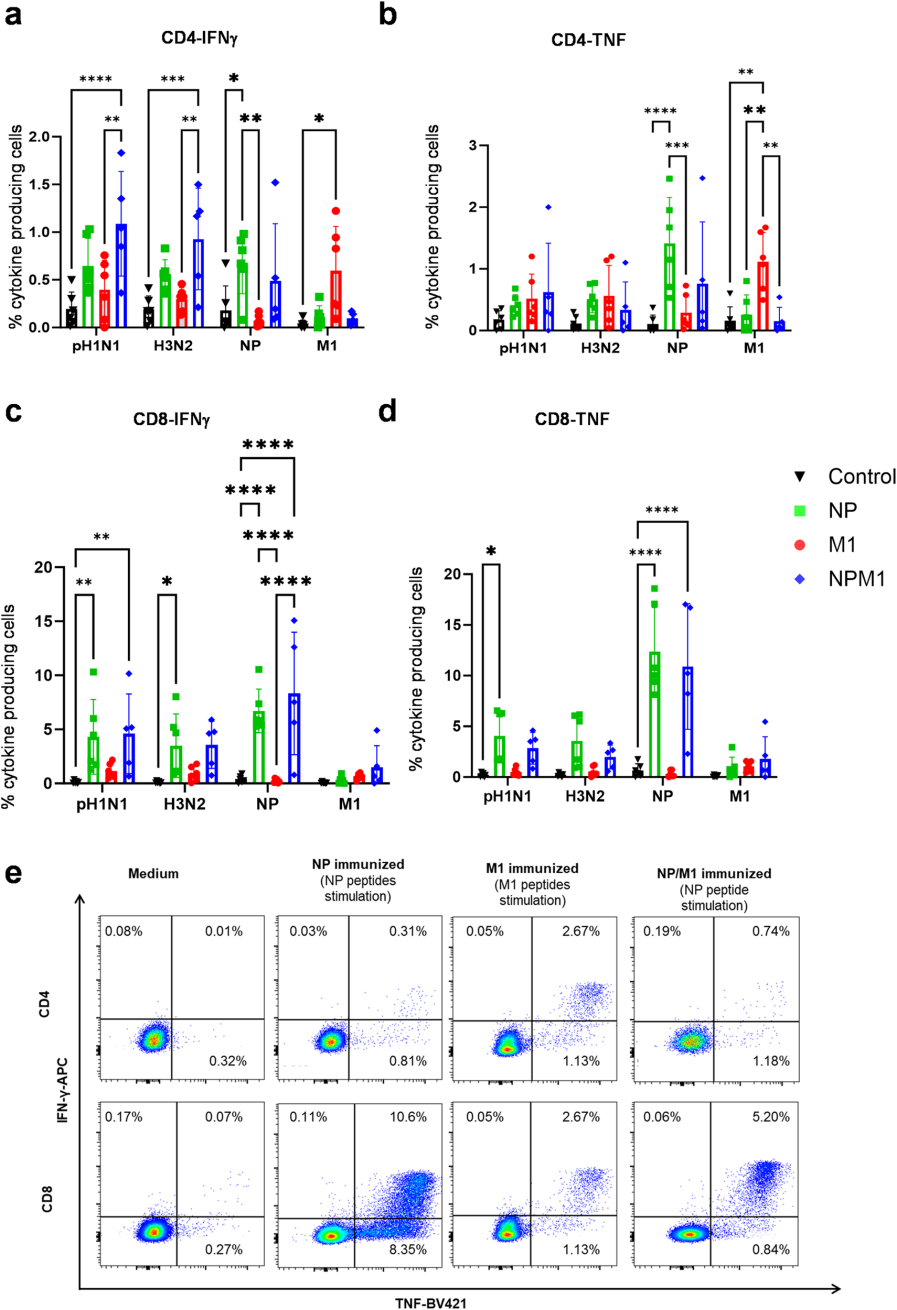
T-cell cytokine responses in BAL. BAL was collected 4 weeks after the MVA-NP, M1 or NPM1 immunization. Cryopreserved cells from D83 were thawed, stimulated with pH1N1, H3N2, NP and M1 and total IFNγ and TNF cytokine secretion was measured in CD4 (**a**, **b**) and CD8 (**c**, **d**) T cells by intracellular cytokine staining. Each symbol represents an individual animal, the top of the bar the mean and the line the standard error (SEM). Two-way ANOVA and Bonferroni’s multiple comparisons test were used to compare responses between groups and asterisks indicate significant differences (*p < 0.05, **p < 0.01, ***p < 0.001, ****p < 0.0001). Representative FACs profiles showing IFNγ and TNF cytokine secretion by CD4 and CD8 cells as determined by intracytoplasmic staining (**e**). BAL cells from NP and NPM1 immunized animals were stimulated with NP peptides. BAL cells from M1 immunized animals were stimulated with M1 peptides.

However, BAL T-cell responses after H3N2 challenge were analyzed by ICS, to assess IFNγ and TNF production by CD4+ and CD8+ T cells (Fig. 5). NPM1 immunization induced the highest frequencies of pH1N1 and H3N2-specific IFNγ producing CD4+ cells, which were significantly higher than the single M1 group. NP-specific CD4+ TNF and CD4+ IFNγ producing cells were highest in the NP immunized group, while M1-specific TNF and IFNγ producing CD4+ responses were highest in the M1 immunized group (Fig. 5a, b).

All immunizations induced much higher frequencies of cytokine producing CD8+ T cells in BAL compared to CD4+ T cells. IFNγ production following pH1N1 or NP stimulation was significantly greater in the NPM1 and NP groups compared to unimmunized controls (Fig. 5c). NP immunization also induced significantly higher H3N2-specific IFNγ response compared to controls (Fig. 5c). M1 immunized animals made IFNγ and TNF producing CD8+ cells following M1 stimulation, but these did not differ significantly from the pH1N pre-exposed animals (Fig. 5e).

In summary, ICS and ELISpot data suggest that NP, M1 and NPM1 immunization induced strong IFNγ pH1N1 and H3N2 virus-specific CD4+ and CD8+ responses within the lung. Strong NP- and M1-specific IFNγ responses were detected by ELISpot and both peptide specific TNF and IFNγ responses were detected by ICS. NP induced a strong CD8+ response following NP stimulation, while M1 induced more balanced CD4+ and CD8+ responses following M1 stimulation.

### Correlations between immune parameters and virological/pathological measures

Correlations were assessed between sixteen immune parameters related to NP and M1 and five virological or pathological measures (H3N2 nasal shedding, gross pathology (Halbur), percentage lung involvement, gross pathology (Morgan) and NP staining (IHC)) using Spearman’s rank correlation coefficient (*ρ*) calculated from data for all pigs.

**Fig. 6:**
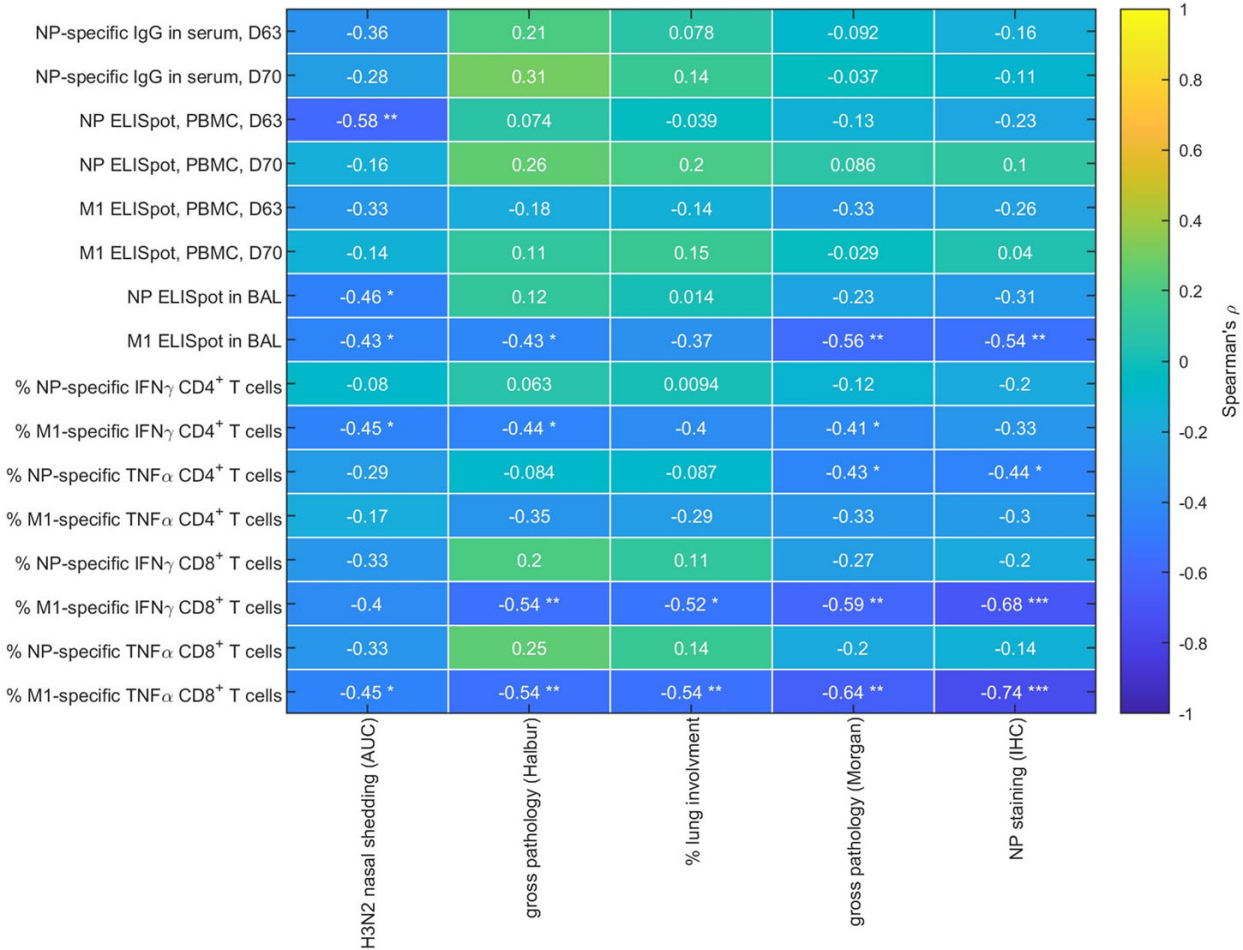
Correlations between immune parameters and virological or pathological measures. The plot shows Spearman’s rank correlation coefficient (*ρ*; indicated by the scale bar) between each immune parameter (row) and virological or pathological measure (column) using data for all four treatment groups (i.e. controls, single M1, single NP and NPM1 immunized pigs). Asterisks indicate correlation coefficients significantly different from zero (*p < 0.05, **p < 0.01, ***p < 0.001).

The percentage of M1-specific TNF-secreting CD8^+^ T cells was negatively correlated with all five virological/pathological measures (−0.74 < *ρ* < −0.45), while the percentage of M1-specific IFN*γ* secreting CD8^+^ T cells and the M1 ELISpot response in BAL were both negatively correlated with four out of five measures (−0.68 < *ρ* < −0.52 and −0.56 < *ρ* < −0.43, respectively) (Fig. 6). In addition, the NP ELISpot response in PBMCs at D63, the NP ELISpot response in BAL, the percentage of M1-specific IFN*γ*-secreting CD4^+^ T cells and the percentage of NP-specific TNF-secreting CD4^+^ T cells were all negatively correlated with at least one virological or pathological measure, using data for all four treatment groups (i.e. controls, single M1, single NP and NPM1 immunized pigs) (Fig. 6). Scatter plots for the strongest correlations in Fig. 6 are displayed in Supplementary Fig. 7.

These negative correlations indicate that higher values of the immune parameter are correlated with lower values of the virological/pathological measure, suggesting a correlation with protection. Although there was no correlation with the NP-specific IgG titers in blood, it is still possible that both systemic and mucosal NP antibody responses may contribute to protection through Fc mediated functions.

## Discussion

The development of BPIV will obviate the need to update seasonal influenza vaccines annually and would provide protection against novel epidemic and pandemic influenza virus strains. Several approaches have focused on the hemagglutinin (HA), including attempts to immunize against conserved epitopes of the HA stem and immunization against multiple HA variants^33–36^. More recently it has been recognized that induction of antibodies against the neuraminidase (NA) may be a useful strategy as this molecule is much less variable than HA^12–19^. In this study, we explored a different strategy, the induction of T cell-mediated responses against conserved internal proteins of influenza, as well as investigating whether a vaccine combining internal proteins and NA, but not HA, is effective.

Using pH1N1 pre-exposed pigs we show that single-dose aerosol immunization with ChAdOx2-NPM1-NA2 significantly reduces lung pathology following H3N2 challenge. There was a trend toward reduced viral shedding which was correlated with increased antibody and T-cell immune responses. Although the reduction in viral shedding and lung pathology was less impressive than that the complete abrogation of viral shedding and lung pathology seen with prime-boost immunizations with ChAdOx2-NPM1-NA2 and MVA-NPM1-NA2^20^, it is conceivable that it might prevent severe and life-threatening disease in humans. Because the vaccine contained the same NA as in the challenge H3N2 virus the mechanism of protection in this study remains unclear. However, the results suggest that this combined internal proteins plus neuraminidase strategy merits further study. Additional investigations will be required to establish if two aerosol doses of ChAdOx2-NPM1-NA2, a more easily deployable regimen will provide protection comparable to the heterologous prime-boost regime of ChAdOx2-NPM1-NA2 and MVA-NPM1-NA2, or whether dose optimization could increase protection after a single dose. An adenoviral vectored vaccine against SARS CoV-2 administered by aerosol has been widely used in China^37–39^. Additionally, aerosol delivery has been utilized for a measles vaccine in Mexico and India^40,41^.

We further evaluated the contribution of NP and M1 in protection in a vaccine containing only internal proteins and were not able to find clear benefits in using both antigens. Although immunization with either NP, M1 or NPM1 provided protection, M1 on its own induced a significant reduction of gross pathology, area of lung involvement, histopathology, and staining for NP within the lung compared to unimmunized controls. M1 immunization did not generate antibody responses but induced both CD4+ and CD8+ M1-specific T-cell responses which correlated with reduced lung pathology or viral load. The lack of detectable antibody response in M1 immunized pigs and the substantial genetic and antigenic differences between the pre-exposure pH1N1 and the challenge H3N2 viruses indicate that the observed protection is likely mediated by mechanisms other than antibodies, almost certainly the T-cell responses detected. In contrast, although NP immunization induced both non-neutralizing antibody responses and T-cell responses, this did not translate into better protection compared to M1. Although NP-induced reduction in lung pathology correlated with stronger T-cell responses (Fig. 6), it is still possible that Fc mediated systemic or mucosal NP antibody functions, which we did not measure, contributed to protection^42^. In our pig model most likely the pH1N1 pre-exposure induces a local lung response which was further boosted by the aerosol delivery of the vaccines with the resulting lung response sufficient to inhibit viral shedding and pathology. It will be important to confirm the role of the lung mucosal response and whether similar protection could be achieved following parenteral/intramuscular administration of NP, M1 and NPM1 vaccines to pre-exposed animals.

As most humans are repeatedly exposed to influenza viruses which induce significant local mucosal T-cell and antibody responses, boosting these by immunization of the respiratory tract would be highly beneficial, especially as these responses tend to wane rapidly after natural infection^9^. However, strain-specific T-cell responses have been observed in children up to 1 year after live attenuated influenza virus (LAIV) vaccination^43^ and influenza-specific lung tissue resident memory cells have been detected in older adults^44^ as well as antigen-specific memory T cells in the blood up to 15 years after infection^45^. These observations offer some hope for generating long-lived cellular immunity to influenza. Blood T-cell responses to M1 and NP can be boosted after heterologous prime-boost with ChAdOx and MVA in humans and were maintained at least up to 18 months in younger people^11^. Immunization with adenovirus expressing influenza nucleoprotein (AdNP) in mice, demonstrated that CD8 T_RM_ in the lungs can be maintained for at least 1 year post vaccination, with continuing in situ proliferation of lung T_RM_ up to 8 months after AdNP adminstration^46^. To fully assess the durability of immune responses induced by respiratory immunization, it will be important to perform immunization challenge studies with a longer interval between immunization and challenge, extending to at least 6 months, although it will be challenging as pigs gain weight rapidly.

A limitation of the present study is that there is no empty vector or irrelevant antigen control to account for the possible non-specific protective effects of the viral vectors. Because of the logistics and challenges of housing many pigs in a high containment facility we only used unimmunized pH1N1 pre-exposed animals as controls. However, in a previous study, we used ChAdOx and MVA expressing Ebola glycoprotein, although the vaccines were administered intramuscularly and challenge was performed with pH1N1^47^. In this study there was no difference in viral load between the unimmunised and Ebola-immunized pigs, while there were differences between these two controls and animals immunized with ChAdOx and MVA expressing NP, M1 and HA. Future studies should include an empty vector or irrelevant antigen control. Once the optimal antigen composition and route of administration are determined, larger group sizes should be used to increase the statistical power.

The matrix protein is a structural protein which encodes at least two proteins M1 and M2. M1 is highly conserved and has been used as a target of BPIV^48^. M1 DNA vaccine provided partial protection against homologous virus challenge in mice^49^, while intranasal immunization with M1 protein vaccine formulated with chitosan provided full protection against homologous virus and partial protection against heterologous virus respectively^50^. The mechanism of protection was thought to be mucosal anti-M1 IgA antibodies and T-cell responses^50^. Influenza NP is crucial for the replication and transcription of the influenza genome. NP is a major target for CD8 + T cells^51,52^. It is highly conserved with over 90% homology between influenza viruses of the same type although differing greatly between A and B viruses. NP-based DNA, viral vectored and recombinant protein vaccines have been evaluated in animal models showing varying degrees of protection^53–55^. However, at least in mice, vaccines based on M1, M2 or NP alone provide less adequate protection than those with two or more influenza antigens^48^.

Many murine CD4+, CD8+ and B cell M1 epitopes have been identified in A/WSN//33 (H1N1) or PR8 viruses^56,57^. A single M1 peptide epitope induced T-cell dependent influenza protection in HLA-A*0201/K^b^ transgenic mice^58^. In human epitopes of NP and M1 (and PB1) are immunodominant targets for cross-reactive T cells and similar epitopes were observed in mapping responses of CD4+ and CD8+ T cells to the entire genome of an H5N1 virus in unexposed individuals^59^. The inclusion of both antigens in the NPM1 group did not induce the strongest peptide specific responses in our study. Nevertheless, having both NP and M1 antigens might be beneficial as it increases the chances of inducing both CD4+ and CD8+ responses in a population with diverse MHC types.

The rare adverse event vaccine-induced immune thrombotic thrombocytopenia (VITT) was associated with intramuscular administration of adenoviral vectored vaccines in the Northern Hemisphere, whereas the use of aerosol delivery and the vaccine might be of particular interest to countries in the global South. Anti-vector immunity does not prevent re-use of the same vector. ChAdOx1 nCoV-19 has been administered to the same individuals four times with boosting seen each time, and a current clinical trial is assessing use of ChAdOx1 MERS in subjects who previously received ChAdOx1 nCoV-19 or an mRNA Covid vaccine (ISRCTN - ISRCTN17936606: A study of a new vaccine against the MERS virus in adults aged 50–70 years). We have previously demonstrated the protective efficacy of NP/M1-NA2 viral vectored vaccines in the pig pre-exposure model^20^. Here we show that there is no advantage in including both NP and M1 antigens, a critical finding for the development of a deployable vaccine. Omitting one of these antigens leaves space to include NA and/or HA in the vaccine to provide strain-specific antibody protection. This addition might complement M1’s or NP’s capacity to provide broader T cell-mediated protection.

We have previously observed differences in vaccine-induced protection between pigs and mice^31,47,60,61^. In pigs, intranasal Ad5-HA1-NP and IL-1beta immunization induced increased NP-specific responses, which were associated with enhanced lung pathology following heterologous H3N2 challenge^60^. In contrast in mice, this regime was highly protective against both homologous and heterologous influenza challenge^62^. Similarly, Heinen et al. showed that immunization of pigs with DNA expressing either M2 or an M2-NP fusion protein exacerbated disease after challenge with H1N1 influenza virus^63^ whereas in mice immunization against M2 was protective. It is clear that memory T-cells resident in the lungs are important in mediating cross-protective immunity while at the same time they contribute to lung pathology^64–66^. Further work is required to understand how to deliver antigens to preferentially induce the right quantity and quality of cells in the respiratory tract to skew the balance toward protection over tissue destruction.

Immunization is the most cost-effective public health strategy to combat influenza since its introduction 60 years ago, but current influenza vaccines are less effective against newly emerging influenza strains. Harnessing heterosubtypic immunity is necessary to develop influenza vaccines protective against a broad range of antigenically distinct influenza strains for both seasonal and pandemic influenza. We provide the first direct evidence that T-cell responses induced by aerosol immunization can be protective in the pig pre-exposure model and that single antigen immunization by M1 is sufficient to induce protective immune response. These findings provide valuable insights into the antigen composition of BPIV in a highly relevant large animal model and warrant the further development of cross-protective T cell-based influenza vaccines.

## Materials and methods

### ChAdOx2 and MVA viral vectored vaccines

The production of ChAdOx2 NPM1, ChAdOx2-NPM1-NA2, MVA-NPM1 and MVA-NPM1-NA2 vaccines has been described previously^32^. The NP and M1 protein ORFs were derived from A/swine/England/1353/2009 (GenBank accession number KR701098 and KR701100) and the neuraminidase (NA2) is from H3N2 strain A/swine/Ohio/A01354299/2017 (GenBank accession number MF801571).

ChAdOx2 expressing single antigens, NP or M1 were generated by a similar method. Briefly, either the NP ORF and linker sequence or M1 ORF and linker sequence were removed from the Gateway® recombination shuttle plasmids used to generate ChAdOx2 NPM1 by inverse PCR and plasmid self-ligation. The resulting shuttle plasmids were used to insert the single antigen expression cassettes into the ChAdOx2 Gateway Destination plasmid by Gateway recombination as previously described^67^. Adenoviruses were generated and titred by Viral Vector Core Facility, University of Oxford.

MVA vaccines expressing single antigens under the control of F11 promoter were generated by inverse PCR followed by self-ligation of the shuttle plasmids used to create MVA-NPM1. The resulting shuttle plasmids were used in generate MVA-NP and MVA-M1 through recombination with wt MVA in vitro by Viral Vector Core Facility, University of Oxford.

### Mouse immunogenicity study

The mouse study was performed in accordance with the UK Animals (Scientific Procedures) Act 1986 and with approval from the relevant local Animal Welfare and Ethical Review Body at the University of Oxford (AWERB) (Project License PP2353929). Female, 6-week-old Balb/C mice were purchased from a Home Office approved breeder and supplier (Envigo RMS, UK LTD, INOTIV, UK). After a week of acclimatization and ensuring animals were over 18 grams of weight, vaccines formulated in endotoxin-free PBS were administrated by a 50μl intramuscular injection (IM) into the *musculus tibialis* of the left hind leg (3 groups, *n* = 5) or by intranasal immunization in the nose through a 30 μl slow drop-dripping pipetting (3 groups, *N* = 5). When feasible, staff performing vaccinations and sample harvesting were blinded to the groups and unblinded when data analysis is performed. Vaccine administration was performed under general anesthesia (Isoflurane, IsoFlo®) with full unconsciousness (pedal withdrawal reflex check) for IM route and lighter depth of anesthesia for IN route to allow deep breaths. Mice were immunized with 10^8^ IU of ChAdOx2 NP, M1 or NPM1 vector vaccines and 4 weeks later boosted with 10^6^ PFU of the same antigen vaccine candidate on MVA vector platform. All animals were humanely culled 4 weeks after the final vaccination by an approved Schedule 1 method: exsanguination via cardiac puncture under general anesthesia followed by cervical dislocation and a cervical dislocation with *a posteriori* exsanguination when lungs were needed to be collected. Once death is confirmed organs were harvested and processed in BSL2 cabinets.

### Pig challenge studies

The pig studies were approved by the ethical review processes at VetQuest and the Pirbright Institute in accordance with the UK Government Animal (Scientific Procedures) Act 1986 under Project Licence PP2064443.

In the first study (ChAdOx2-NPM1-NA2 alone), twelve 6-week-old female Landrace x Large White pigs were divided into two groups of six. The pigs were screened by ELISA for the absence of serum IgG titers against pH1N1 and H3N2 and after a week of acclimatization, all pigs were challenged intranasally (IN) with 5 × 10^6^ PFU of A/swine/England/1353/2009 (pH1N1) MDCK grown virus in a total of 4 ml (2 ml per nostril) using a mucosal atomization device (MAD, Wolfe-Tory Medical). Following the inoculation, daily nasal swabs were obtained for 1 week to assess viral load by plaque assays and blood was collected weekly. Twenty-four days following the pH1N1 inoculation, the pigs from one group were sedated by intramuscular injection with 3 mg/kg azaperone (Stresnil, Elanco, UK) and 1.5 mg/kg tiletamine/zolazepam (Zoletil, Virbac, France) and were immunized by aerosol with 5 × 10^8^ infectious units (IU) of the ChAdOx2-NPM1-NA. For the immunization, 1 mL of the vaccine diluted in PBS was administered over 2–5 min using an Aerogen Solo vibrating mesh nebulizer (Aerogen, Dangan, Galway, Ireland). This route of administration reaches both the upper and lower respiratory tract as previously shown using scintigraphy^30^. The unimmunized group served as the Control (C). Four weeks later, all 12 pigs were infected intranasally with 2 mL per nostril of 1.2 × 10^8^ pfu of A/swine/Ohio/A01354299/2017 (H3N2) MDCK grown virus. Daily nasal swabs were collected to assess viral shedding as described above, and 4 days later all pigs were euthanized. The pigs were culled using intravenous pentobarbital (Dolethal 200 mg/ml) and after confirmation of permanent cessation of circulation, PBMC, lung and bronchoalveolar lavage (BAL) were collected to assess viral load, lung pathology and immune responses as previously described^20^.

For the second study to assess the contribution of NP and M1 antigens twenty-four 6-week-old female influenza-free Landrace x Large White pigs were infected intranasally with 3 × 10^6^ PFU of A/swine/England/1353/2009 (pH1N1) MDCK grown virus in a total of 2 mL (1 mL per nostril). Daily nasal swabs were collected for 1 week to assess viral load following H1N1 infection. Four weeks after pH1N1 exposure pigs were randomly divided into four groups of six animals. Three groups were sedated and immunized by aerosol with either (A) ChAdOx2-NPM1, (B) ChAdOx2-NP or (C) ChAdOx2-M1. Each vaccine was administered at 5 × 10^8^ IU in 1 mL using an Aerogen Solo vibrating mesh nebulizer. The last group of six animals did not receive any vaccine and served as control. Four weeks after the first immunization, the pigs were boosted by the same route with the same antigen 1.5 × 10^8^ PFU each of MVA-NPM1, MVA-NP or MVA-M1. The control animals were pH1N1 infected but unimmunized. Four weeks later, all pigs, including the control group, were infected intranasally with 5.7 × 10^8^ pfu of A/swine/Ohio/A01354299/2017 (H3N2) MDCK grown virus in a total of 4 mL (2 mL per nostril) using a MAD. Daily nasal swabs were collected to assess viral shedding. Four days later the animals were euthanized, lung pathology was assessed and PBMC and BAL were collected to investigate immune responses. Viral load was assessed by plaque assays as previously described^20^. Gross and histopathological analyses were performed as described in Vatzia et al.^20^.

### ELISA

Endpoint ELISA for pH1N1, H3N2 and recombinant NA protein from H3N2 A/swine/Ohio/A01354299/2017 (NA2) (sequence matched to the vaccine antigen, Genbank accession number: ATE49827, produced by The Native Antigen company) were performed for IgG in serum and IgG and IgA in BAL as described before^20,32^. NP ELISA was performed using a commercially available kit ID Screen Influenza A Nucleoprotein Swine Indirect (Innovative Diagnostics). Briefly, sera samples alongside with manufacturer provided positive and negative controls were diluted in the provided buffer and incubated with NP-coated plates in triplicate for 1 h at 37 °C. The plates were washed twice and incubated with HRP conjugated anti-pig antibody and developed using TMB substrate. End point titer was calculated following manufacturer calculation: Log10 end point titer = 1.2* Log10 (S/P) + 3.5, where S/P ratio = Test Sample OD/positive sample OD.

### Murine IFNγ ELISpot and intracellular cytokine staining

Fresh mouse splenocytes and lung cells were used for ELISpot and ICS (Spectral flow cytometry). Spleen and lung cell suspension were prepared using C tubes and gentle MACS dissociator. Lungs homogenates were incubated in collagenase solution (Collagenase 0.7 mg/ml, Sigma; DNase, 0.03 mg/ml, Sigma) and stopped by adding fetus bovine serum. Homogenates were filtered through a 70 µm cell strainer and red blood cells were lysed with ammonium chloride lysis solution prior to resuspension in complete media (α-MEM, 10%FBS, 1%Pen/Strep, 1% L-Gln). Cell suspensions were stimulated with pool of 15 mer peptides overlapping by 11 (Mimotopes), spanning the length of the proteins NP and M1 as previously described^20,32^. For ELISpots, cells and peptides were added to Hydrophobic-PVDF ELISpot plates (Merck) coated with 5 µg/ml of anti-mouse IFNγ (AN18, Mabtech). After 18–20 h of stimulation at 37 °C, IFNγ spot forming cells (SFC) were detected with anti-mouse IFNγ biotin (R46A2, Mabtech) followed by streptavidin-alkaline phosphatase (Mabtech) and development with AP conjugate substrate kit (Bio-Rad, UK). Spots were counted using an AID ELISpot reader and software (AID).

For ICS mouse splenocytes and lung cells, cells were stimulated at 37 °C for 5 h with 2 µg/ml peptide pool. Media and PMA positive control was included. A cocktail of 1 mg/mL GolgiPlug (BD) and CD107a-A647 (Clone 1D4B) was added to the stimulation cocktail. Viability of cells was detected by staining with Zombie NIR (BioLegend). Following surface staining with CD3-A700 (Clone 17A2) CD4-BUV496 (Clone GK1.5, BD), CD8-BV570 (Clone 53-6.7), CD11a-PECy7 (Clone H155-78), CD4-BV785 (Clone IM7), CD62L-PECF594 (Clone MEL-14), CD69-PECy5 (Clone H1.2F3), CD103-PerCPCy5.5 (Clone 2E7), CD19-BUV737 (Clone 1d3) and CD4 5-APCFire750 (Clone 30F11) diluted in Brilliant Stain Buffer (BD), cells were fixed with 4% paraformaldehyde and stained intracellularly with IL2-PE (Clone JES6-5H4, Thermo Fisher Scientific), IL4-BV711 (Clone 11B11), IL10-BV605 (Clone JES5-16E3), IFNg-BV421 (Clone XMG1.2) and TNF-A488 (Clone MP6-XT22) diluted in Perm-Wash buffer (BD). Unless stated all antibodies purchased from BioLegend. Sample acquisition was performed on an ID7000TM Spectral Cell Analyzer and data analyzed in ID7000 analysis software and FlowJo V10 (TreeStar). Antigen-specific T cells were identified by gating doublet negative (FSC-H vs FSC-A and SSC-H vs SSC-A), size (FSC-A vs SSC), on LIVE/DEAD negative, CD45+, CD3+, CD4+ or CD8+ cells and each individual cytokine. T cell subsets were gated within the population of IFNγ and/or TNF responses and are presented after subtraction of the background response detected in the corresponding media-stimulated control sample for each mouse. CD8+ tissue resident memory (TRM) cells in lungs were identified as CD44hi CD69+CD103+ cells (Supplementary Fig. 4).

### Porcine IFNγ ELISpots and intracellular cytokine staining

Cryopreserved porcine BAL cells and PBMC were used to assess the frequencies of IFNγ-producing cells by ELISpot as described before^32^. The BAL T-cell responses were also assayed by intracellular cytokine staining (ICS). IFNγ, TNF and IFN/TNF production by CD4+ and CD8+ β T cells was measured following pH1N1, H3N2, NP1, NP2 and M1, stimulation (gating strategy shown in Supplementary Fig. 6) as previously described^32^. Briefly, BAL cells were either stimulated overnight with live pH1H1 or H3N2 virus (MOI = 1) or for 5 h with peptide pools covering NP and M1 proteins (2 μg/ml) and were incubated at 37 °C with 5% CO_2_. The peptide sequences have been published previously^32^. One hour after the start of peptide stimulation, Brefeldin A (GolgiPlug™, BD Biosciences) was added. Phorbol 12-myristate 13-acetate (PMA)/ionomycin (BioLegend) was added as a positive control at the same time as the GolgiPlug. Four hours later, the cells were centrifuged for 4 min at 1500 rpm, washed twice with PBS and stained with the primary antibodies CD4-PerCP-Cy5.5 (74-12-4, BD Biosciences), CD8β-FITC (PPT23, Bio-Rad Laboratories) and the Near-Infrared Fixable LIVE/DEAD stain (Invitrogen) for identification of the live cells. Twenty minutes after the surface staining and incubation at 4 °C, the cells were fixed and permeabilized with BD Cytofix/Cytoperm (BD Biosciences) and were then stained and incubated for 30 min at 4 °C with the anti-cytokine antibodies TNF-BV421 (MAb11, BioLegend) and IFNγ-APC (P2G10, BD Biosciences). Finally, the cells were washed twice, resuspended in PBS, acquired using a MACSquant Analyzer 16 (Miltenyi) and analyzed by FlowJo software version 10.8 (FlowJo LLC, Ashland, OR, USA). The frequency of cytokine production shown is after subtraction of the frequencies found in medium control wells (unstimulated cells that were also seeded overnight and treated with Brefeldin A as described above).

### Statistical analysis

GraphPad Prism 9.2.0 (GraphPad Software, San Diego, CA, USA) was used for all statistical analyses except for the correlation analysis. The data sets were first analyzed for normality and then subjected to either an unpaired *t* test when only one time point for two groups was compared (PM day for BAL and PBMC) and the data were normally distributed or to a Mann–Whitney test when the data were not normally distributed (pig experiment 1). When multiple time points were included (ELISAs pig experiment 1 time-course and all analyses for experiment 2) the data were first analyzed for normality and then subjected to a two-way-ANOVA and Bonferroni’s multiple comparisons test if normally distributed or to a Kruskal–Wallis test and Dunn’s multiple comparisons test when normality was not achieved. Significant differences were either presented on each graph or listed in the figure legends (*p < 0.05, **p < 0.01, ***p < 0.001). To explore the correlation between immune parameters (16 in total) and virological/pathological measures (5 in total) Spearman’s rank correlation coefficient (*ρ*) was computed for each pair of measures using data for all four treatment groups (i.e. controls, single M1, single NP and NPM1 immunized pigs). This analysis was implemented in Matlab (version R2020b; The Mathworks Inc.).

## Supplementary information

Supplemental Information

## Data Availability

Data generated or analyzed during this study that are critical to the reported findings are available within the article and its Supplementary Information files. Additional supporting data are available from the corresponding authors without undue reservation.
