# Supplementary material for: Aerosol immunization with influenza matrix, nucleoprotein, or both prevents lung disease in pig
Source: NPJ Vaccines. 2024 Oct 13;9:188. doi: 10.1038/s41541-024-00989-8 (PMC11471855; doi:10.1038/s41541-024-00989-8)
Supplement: Supplementary file 1 — Supplemental Information [file 41541_2024_989_MOESM1_ESM.pdf]

Suppl. Figure 1

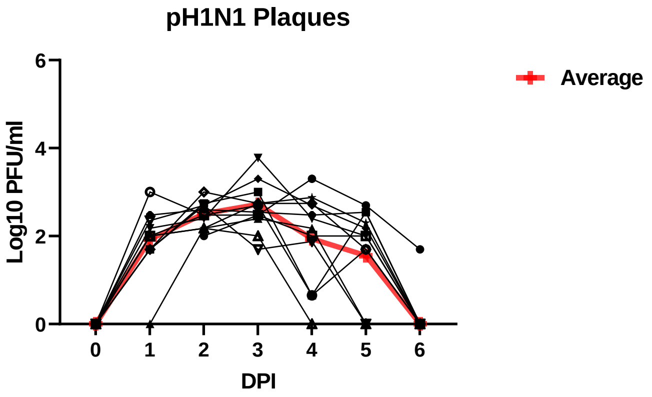

Suppl. Figure 1. Viral load after pH1N1 inoculation and single dose ChAdOx-NPM1-NA immunization. Virus load after pH1N infection was determined by plaque assay of daily nasal swabs at the indicated days post pH1N1 infection (DPI) to confirm successful infection of all animals. The animals were subsequently immunized with a single dose of ChAdOx-NPM1-NA.

Suppl Figure 2

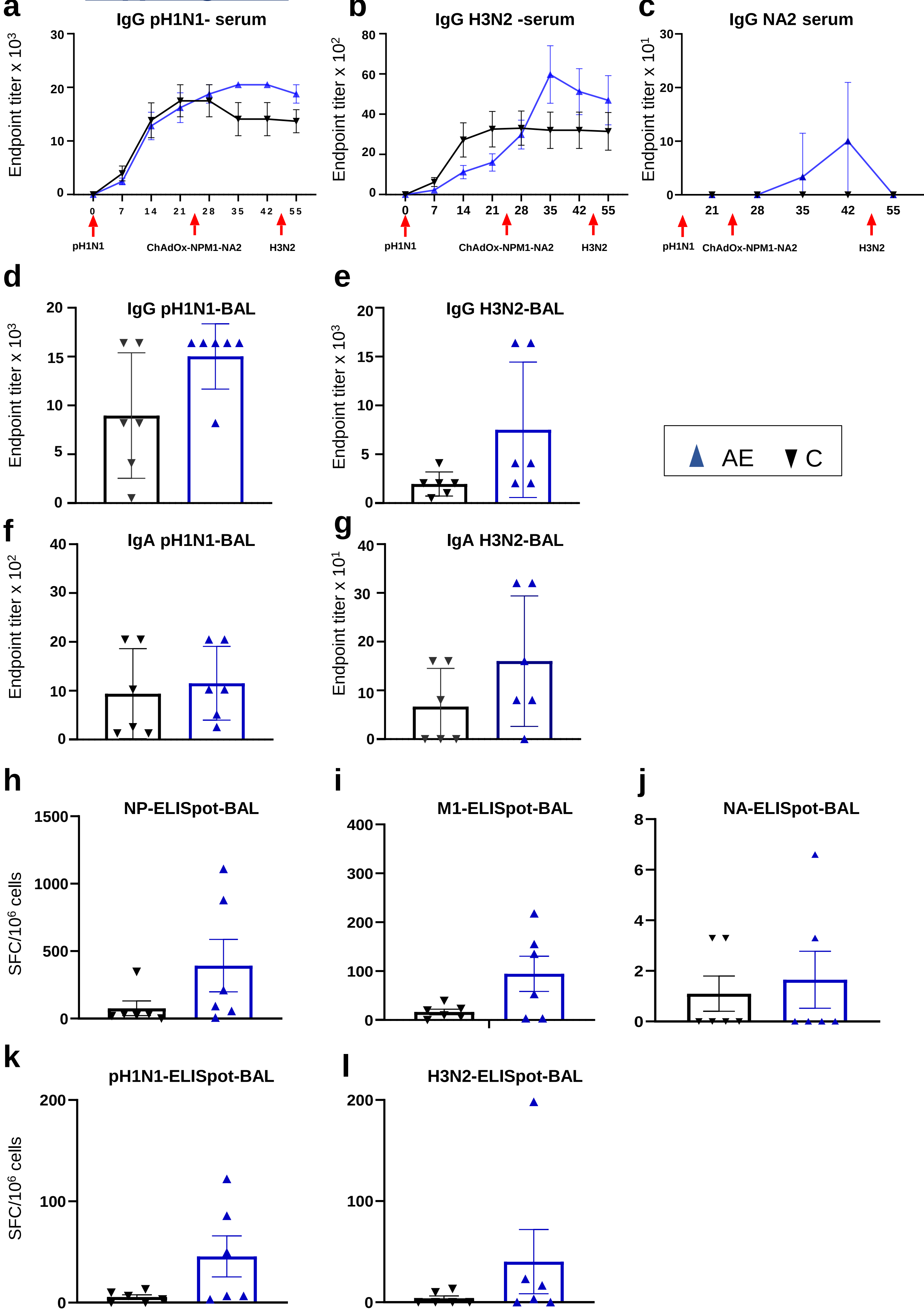

Suppl Figure 2. Antibody and T cell responses following single dose ChAdOx-NPM1-NA2 aerosol immunization. a) pH1N1, **b)** H3N2 and **c)** NA2 specific IgG responses in serum were determined by ELISA at the indicated time points. **d)** pH1N1, **e)** H3N2 specific IgG and **f)** pH1N1 and **g)** H3N2 IgA responses in BAL, were determined by ELISA four days after the H3N2 challenge. The mean and standard error (SEM) is presented in each time point (**a-c) or** at day 52 (PM**) (d-g)**. The arrows below D0, D24 and D48 indicate the time of pH1N1challenge, immunization with ChAdOx-NPM1-NA2 and challenge with H3N2. IFNγ secreting spot forming cells (SFC) were enumerated in BAL **(h-l)** on D52. Cells were stimulated with a pool of peptides covering **h)** NP , **i)** M1 and **j)** NA proteins or **k)** pH1N1 and **l)** H3N2 viruses. Each symbol represents one animal. The top of each bar indicates the mean and the line the standard error mean (SEM) **(d-l).** Data were analyzed either by one-way ANOVA and Bonferroni's multiple comparisons test when the data were normally distributed **(a-c**) or with Kruskal-Wallis and Dunn's multiple comparisons test when normality was not achieved (**d-l)**) .

Supplementary Figure 3

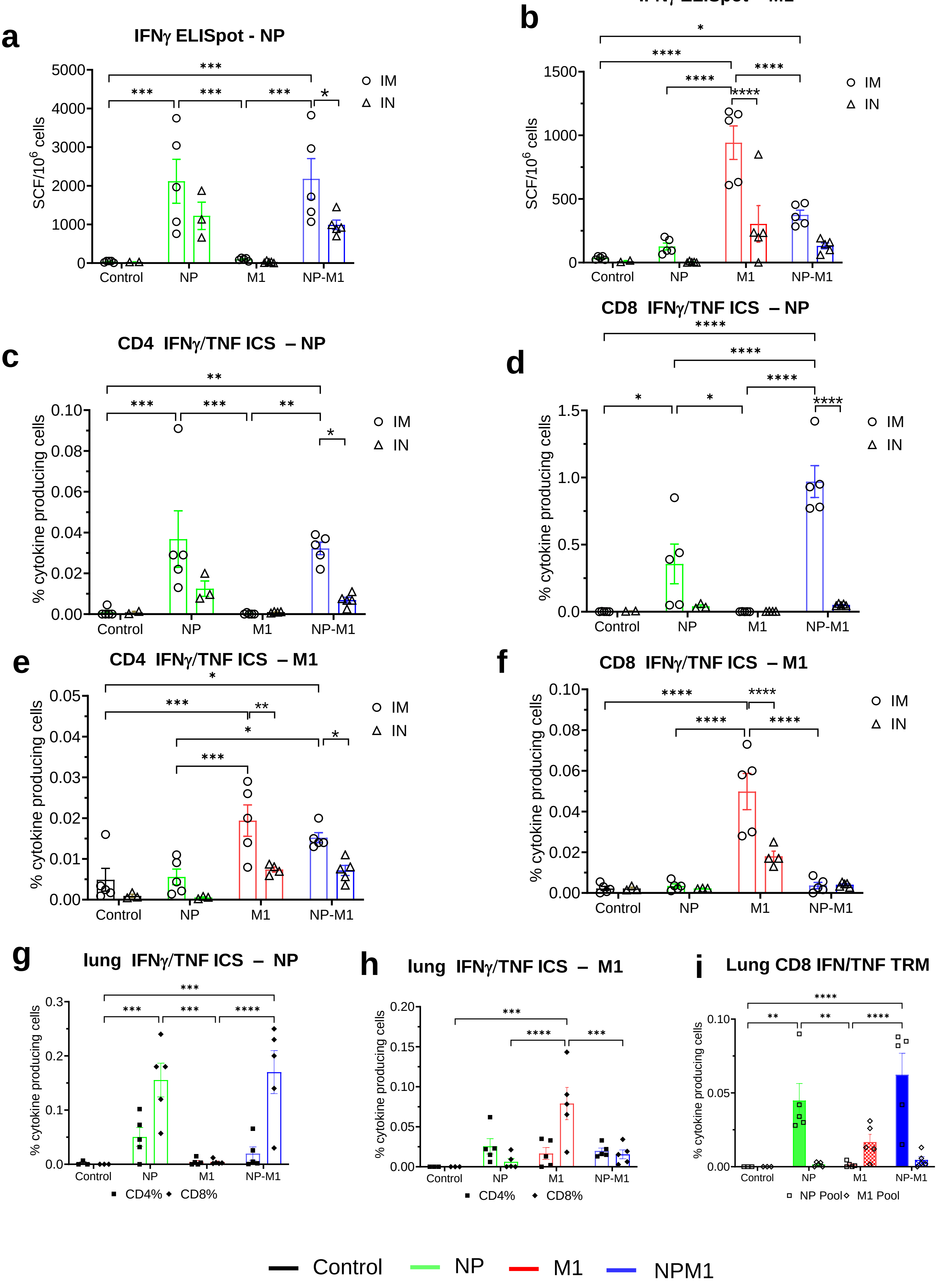

Supplementary Figure 3. IFGγ ELISPOT responses and T cell cytokine responses in immunised mice. Cytokine responses were measure 4 weeks after heterologous prime (ChadOx2) and boost (MVA) of the corresponding vaccines containing NP, M1 or NPM1. The number of IFNγ producing cells after NP (**a**) and M1 pools of peptides (**b**) stimulation of fresh splenocytes of either Intramuscular (IM-o) or Intranasal (IN-Δ) immunisations. Frequency of CD4 (**c**,**e**) and CD8 (**d**,**f**) T cells secreting double IFNγ/TNF cytokines was measured by intracellular staining (ICS) in spleen (**c-f**) and lungs (**g-i**). Fresh splenocytes from immunised mice (IM-o or IN-Δ) were stimulated with NP (**c**,**d**) and M1 (**e**,**f**) pool of peptides**.** Lung cells from intranasally immunised mice were stimulated with either NP (**g**) or M1 (**h**) peptides and percentage of either CD4 (■) and CD8 (♦) IFNγ/TNF double producing cells was obtained by Spectral Flow Cytometry. **i**) Frequencies of NP and M1-specific CD8 IFNγ/TNF producing T resident memory (TRM) cells in lungs of intranasally immunised mice after stimulation with NP (□) or M1 (◊) pools of peptides, is shown. Each symbol represents an individual animal, the top of the bar the mean and the line the standard error (SEM). Two-way ANOVA and Bonferroni's multiple comparisons test were used to compare responses between groups and asterisks indicate significant differences (*p<0.05, **p<0.01, ***p<0.001, ****p<0.0001).

Supplementary Figure 4

a

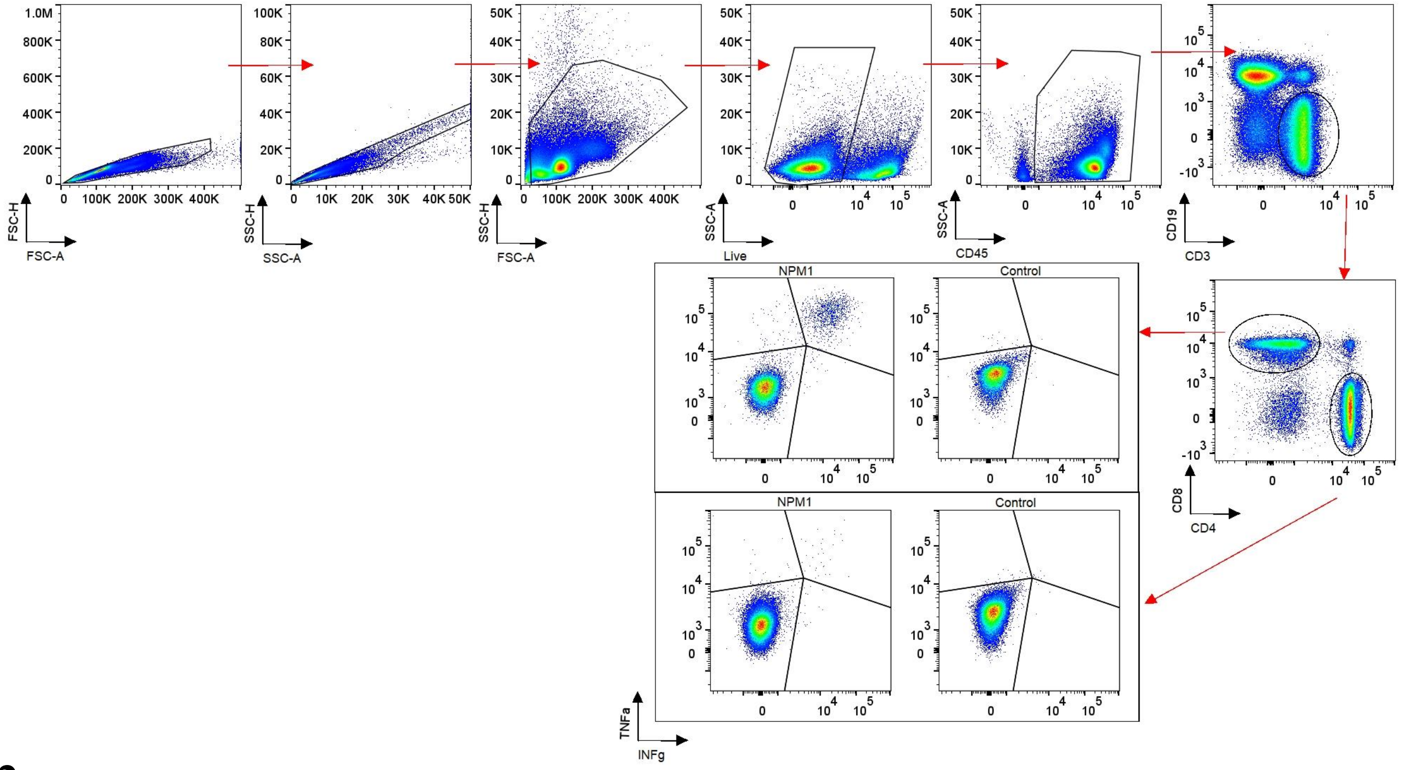

b

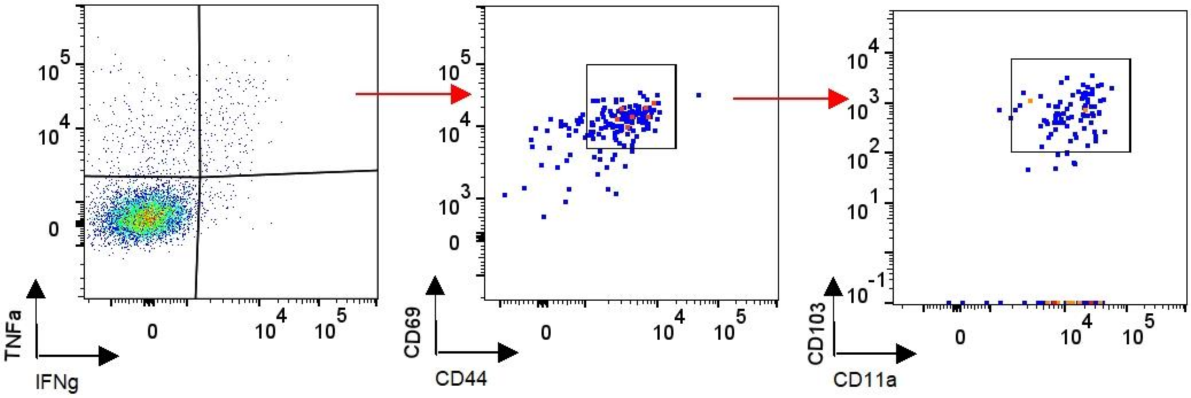

Suppl. Figure 4. Gating strategy for analysis of cytokine production by mouse splenocytes and lung cells. Cells were stimulated for 5 hours with M1 or NP pools of peptides followed by intracellular cytokine staining. Lymphocytes were gated by exclusion of doublets, light scatter properties and exclusion of dead cells. Live cells were gated for CD45+, CD3+, CD4+ and CD8+ cells and production of TNF, IFNγ, TNF/IFNγ was determined with the indicated gates. **a)** Gating strategy for IFNγ and TNF ICS in representative mouse spleen sample after NPM1 prime boost immunization. **b) T**issue resident memory (TRM) cells gating strategy for CD8+ TNF+ IFNγ + CD44hi CD69+ CD103+, in a representative mouse lung sample after prime-boost NPM1 immunization.

Supplementary Figure 5

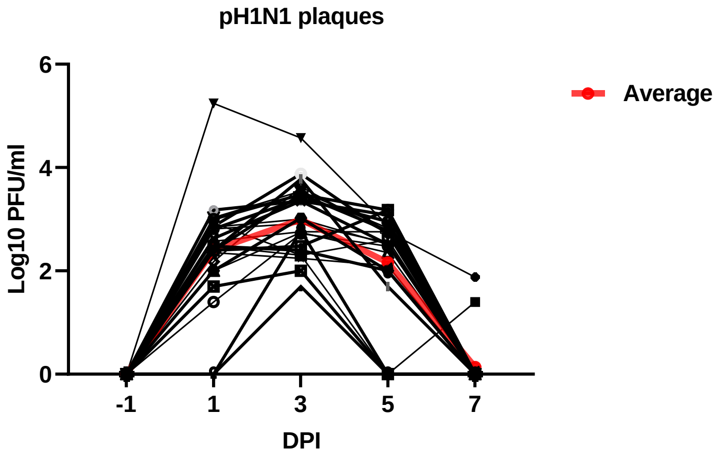

Suppl. Figure 5. Viral load after pH1N1 inoculation. Virus load after pH1N infection was determined by plaque assay of daily nasal swabs at the indicated days post pH1N1 infection (DPI) to confirm successful infection of all animals. The animals were subsequently immunized with ChAdOx2 and MVA containing NP, M1 and NPM1 antigens.

Supplementary Figure 6

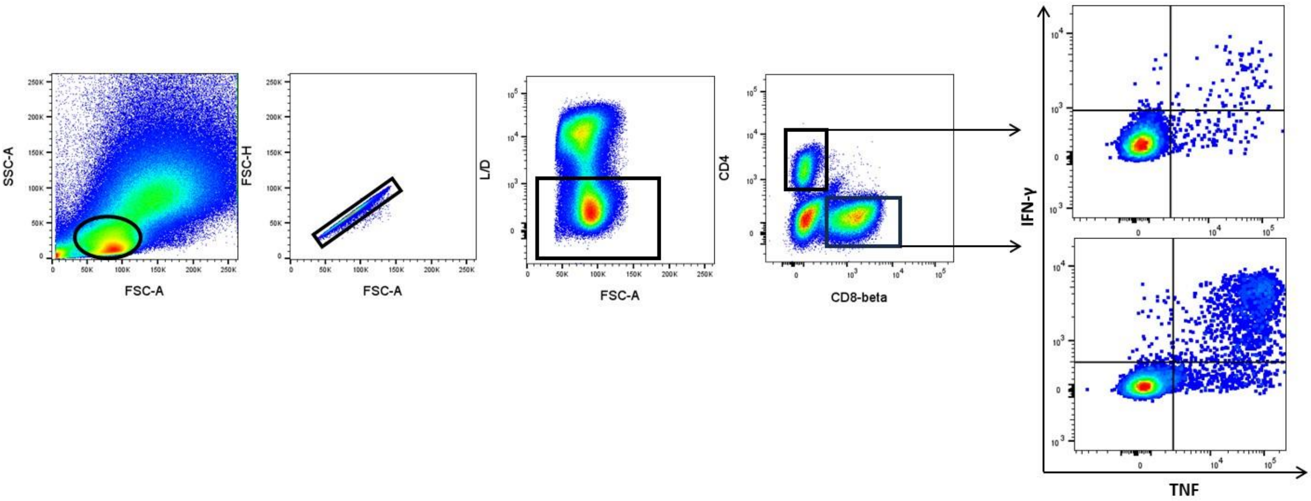

Suppl. Figure 6. Gating strategy followed for analysis of cytokine production by porcine CD4 and CD8 T cells. Cells were stimulated overnight with pH1N1 or H3N2 or for 5 hours with M1, NP or NA peptides followed by intracellular cytokine staining. Lymphocytes were gated by light scatter properties and were further sub-gated for exclusion of doublets and dead cells. Live cells were gated for CD4 and CD8β cells and production of TNF, IFNγ, TNF/IFNγ was determined with the indicated gates. The gates shown are from BAL tissue stimulated with pH1N1.

.

Supplementary Figure 7

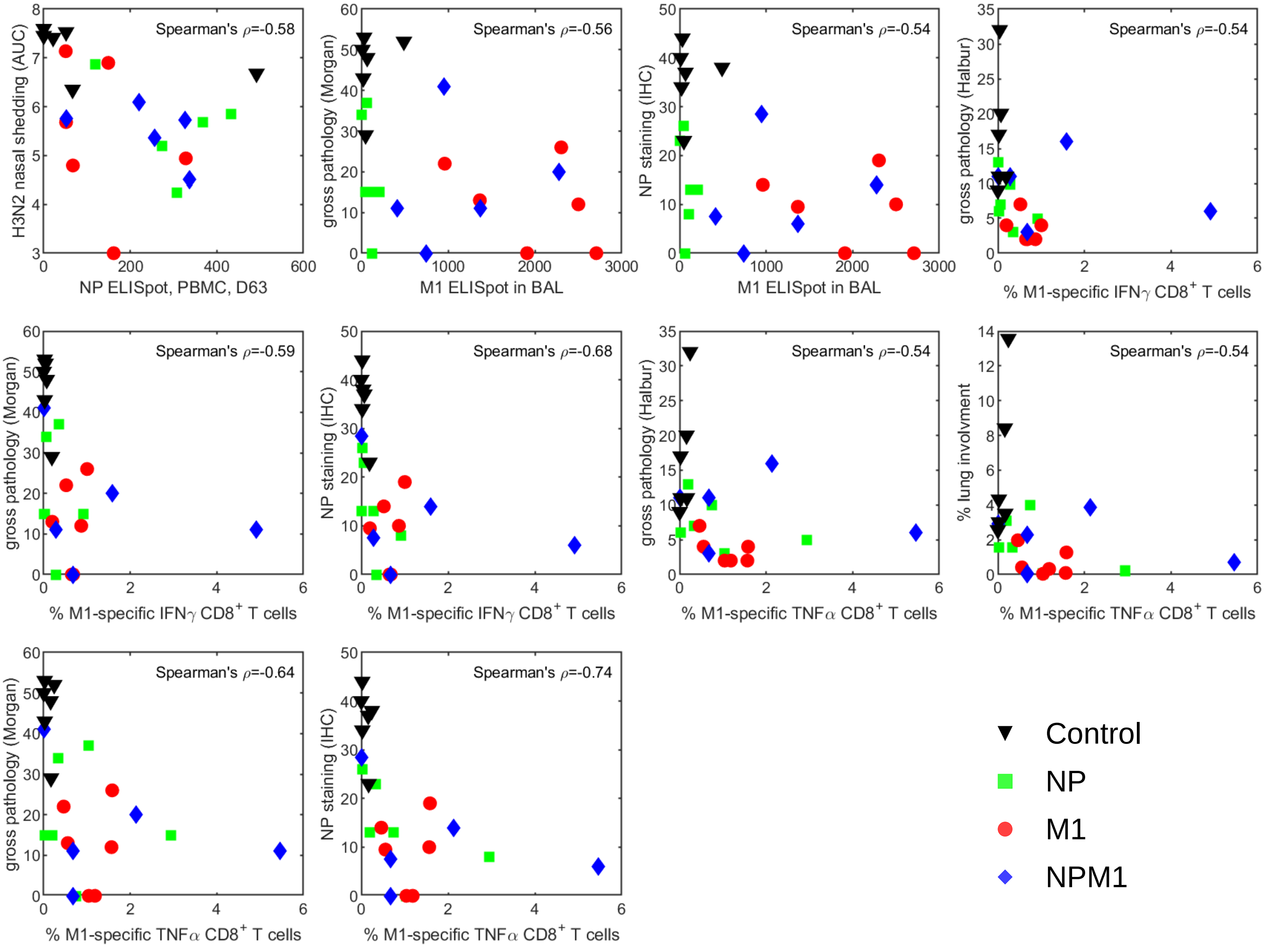

Supplementary Figure 7. Correlations between immune parameters and virological or pathological measures. The parameters and measures plotted are those for which the Spearman's ρ<-0.5 (see Figure 6 in the main paper). Each plot show the immune parameter (on the x-axis) and virological or pathological measure (on the y-axis) for different groups.
